# A Scoping Review of Radiation Oncology Educational and Career-Planning Interventions in Undergraduate Medical Education

**DOI:** 10.3390/curroncol28010072

**Published:** 2021-01-31

**Authors:** Andrew J. Arifin, Karina Liubchenko, Gabriel Boldt, Timothy K. Nguyen

**Affiliations:** 1 Department of Radiation Oncology, London Regional Cancer Program, London, ON N6A 3W9, Canada; Andrew.Arifin@lhsc.on.ca (A.J.A.); Gabriel.Boldt@lhsc.on.ca (G.B.); 2 Schulich School of Medicine & Dentistry, Western University, London, ON N6A 5C1, Canada; kliubche@uwo.ca

**Keywords:** radiation oncology, medical education, medical student, undergraduate medical education, clinical clerkship, mentoring

## Abstract

Radiation oncology (RO) teaching in undergraduate medical education (UME) is lacking worldwide with potentially detrimental effects on medical student career choices and patient care. The objective of this scoping review is to examine the extent of published literature describing RO educational and career-planning interventions in UME. Online databases were searched from respective dates of inception to June 2020 for articles that reported outcomes from RO educational and career-planning interventions in UME. Two independent reviewers screened entries for inclusion. Following full-text reviews, 25 articles were analyzed. Most interventions were a single session, involved clinical medical students, and were based in North America. Didactic teaching was most commonly used, though a majority included interactive learning in addition to or in place of didactic teaching. As expected, there was a heterogeneity of outcomes reported, and most studies collected data using surveys alone. Recurring topics included the multidisciplinary nature of oncology and psychosocial oncology. There was a paucity of studies reporting on formal mentorship programs and research programs. The data collated in this study can help develop new initiatives based on what has succeeded in the past. Areas that may benefit from future studies include mentorship programs, research programs, and interventions from outside North America.

## 1. Introduction

More than 1.8 million new cancer cases are estimated to be diagnosed in 2020 in the United States (US) alone [1]. Radiation oncologists play a pivotal role in the multidisciplinary management of many cancers, as radiotherapy is indicated in an estimated 52% of patients with cancer [2]. However, there is a lack of radiation oncology (RO) teaching in medical school. A survey of final-year medical students in Canada showed that one in five students did not receive any RO teaching, and 65% received less than 2 h of teaching [3]. In a survey of RO departments in the US, 25% of departments described no involvement in formal curricula at their affiliated medical school [4]. Similarly, disappointing results were found across Europe [5]. 

A recent multicenter survey of medical students and primary care physicians in the US revealed that they had a poor understanding of the characteristics of a radiation oncologist, indications for radiation therapy, and related toxicity [6]. These gaps in knowledge among practicing physicians can have detrimental effects on patient care, such as inappropriate referrals that lead to delayed or a lack of care. As physicians of all disciplines may care for patients with cancer, there is a general consensus that all physicians should have familiarity with oncologic treatments, including radiotherapy, and, consequently, this should be addressed during undergraduate medical education (UME) [7]. The extent of RO teaching in UME has been previously reviewed [7]. The authors identified 7 articles that described RO teaching for medical students, though few reported outcomes. Over the last decade, since that report was published, several new teaching interventions have been described and an update on the subject is needed. Furthermore, the extent of published RO programming for UME outside of teaching (e.g., research training, mentorship, career planning) has not been formally reviewed to our knowledge. Herein, we refer to these types of interventions as RO initiatives.

The lack of exposure to RO in medical school may influence the career choices of graduating medical students. It has been shown that exposure to a specialty during medical school correlates with specialty interest across multiple specialties, including RO [3,8]. The impact of RO initiatives on medical student career decisions is not well understood.

The purpose of this scoping review was to examine the extent of published literature describing RO initiatives in UME. Given the anticipated heterogeneity of the included studies and the broad nature of our research topic, a scoping review was chosen over other types of reviews to present a high-level overview of available evidence, to examine the outcomes measured, and to identify areas that may benefit from further research.

## 2. Materials and Methods

Our protocol was developed using guidance from the Joanna Briggs Institute methodology for scoping reviews [9] and the preferred reporting items for systematic reviews and meta-analyses (PRISMA) extension for Scoping Reviews, and revised by the research team. The protocol is available upon request.

PubMed, EMBASE, and Cochrane databases were searched from their respective dates of inception to 3 June 2020. A search strategy was developed by an experienced medical librarian. The complete search strategy is as follows:

- PubMed (180 results): (students, medical[mh] OR clinical clerkship[mh] OR education, medical, undergraduate[mh] OR medical student*[tw] OR undergraduate[tw] OR clerk*[tw]) AND (radiation oncology[mh] OR radiation oncology[tw] OR brachytherapy[tw] OR radiotherapy[tw] OR radiotherapy[mh] OR radiosurgery[tw] OR radiosurgery[mh] OR stereotactic[tw] OR SBRT[tw] OR SABR[tw])
- OVID Medline, Cochrane (363 results): (exp medical student/ or exp clinical education/ or exp medical education/ or exp medical student/ or medical student*.mp. or undergraduate.mp. or exp undergraduate student/ or clerk*.mp.) and (radiation oncology.mp. or exp radiation oncology/ or exp radiotherapy/ or radiotherapy.mp. or exp brachytherapy/ or brachytherapy.mp. or radiosurgery.mp. or exp radiosurgery/ or stereotactic radiosurgery/ or stereotactic.mp. or sbrt.mp. or exp stereotactic body radiation therapy/ or sabr.mp.)

Search results were imported into spreadsheet software for sorting. Inclusion criteria were developed a priori, and included peer-reviewed, English-language articles describing a RO education intervention or RO initiative involving undergraduate medical students in which an outcome is reported. Qualitative and mixed-method studies were included. Two independent reviewers were involved with study selection with a third available to settle discrepancies. The screening process was performed twice: the first using titles and abstracts only, and the second using the full text of each publication. 

Data abstraction was performed by one reviewer and verified by another. The data charting form was refined throughout the review process and information updated in an iterative process.

Abstracted data included demographic characteristics (e.g., number of participants, number of survey respondents, level of training, geographic location), intervention characteristics (e.g., type, duration, objective), study characteristics (e.g., methodology, comparators, statistics), and descriptions of outcomes. Recurring themes were noted in a narrative comments section of the data abstraction form.

Consistent with guidance for scoping reviews, we did not perform a critical appraisal of individual sources for methodological quality or risk of bias.

To summarize categorical data of studies, descriptive statistics and frequency analyses were performed. Quantitative data from studies that reported test scores and career choices of participants were collected and summarized. Studies were grouped by intervention objective and summarized by demographic information, intervention type, methodology, and outcomes. 

## 3. Results

**Figure 1:**
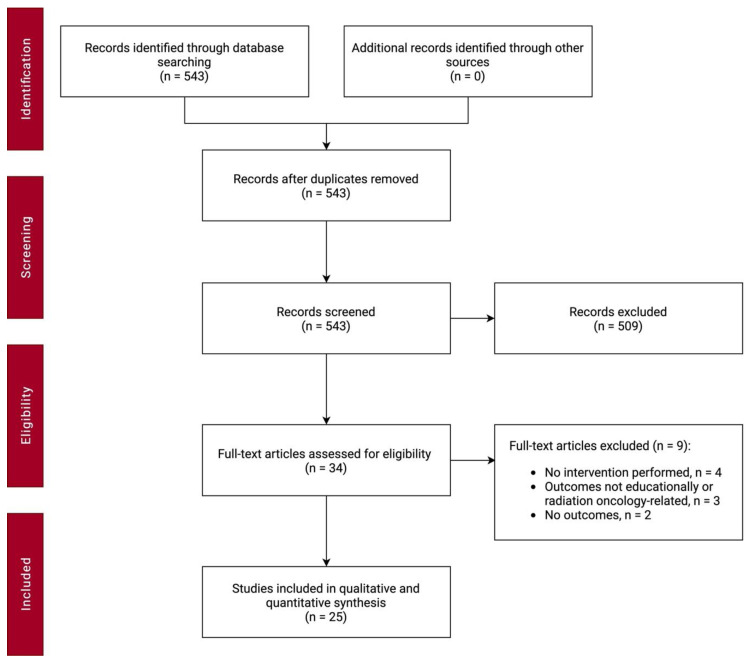
Preferred reporting items for systematic reviews and meta-analyses (PRISMA) flow diagram.

A PRISMA flow diagram describing the screening process is detailed in Figure 1. The initial search of databases identified 543 records. Study titles and abstracts were screened, and 509 records were excluded. Full-text review was performed on 34 articles, following which, 25 articles were included for qualitative synthesis [10,11,12,13,14,15,16,17,18,19,20,21,22,23,24,25,26,27,28,29,30,31,32,33,34]. Table 1 summarizes the review findings by data type. A summary of individual studies grouped by intervention objective can be found in Table S1.

### 3.1. Demographic Characteristics

The target populations studied were clinical (i.e., third- and fourth-year) medical students (n = 12), pre-clinical (e.g., first- and second-year) medical students (n = 8), and all undergraduate medical students (n = 3). The median number of participants was 96.5 (range 16–682), and the median survey response rate was 93.4% (range 53.8–100%). The majority of studies were based in North America (n = 21).

### 3.2. Intervention Characteristics

Most interventions were focused on RO teaching (n = 12) and delivered as a single session (n = 15) in the format of a half-day course (n = 13). Eight studies incorporated an examination to assess knowledge. Didactic lectures were most commonly used (n = 17), although 13 studies employed a component of interactive learning in addition to or in place of didactic learning.

Interactive components included contouring modules, board games, role-playing, and clinical electives. One study described a session where medical students role-played as specialists and patients in a multidisciplinary consultation meeting discussing breast cancer management [12]. The session was followed by a debriefing exercise led by faculty with a focus on the discussion of psychosocial issues during breast cancer treatment. Another described a computer-assisted board game in which players advanced through various specialty clinics, mimicking a patient’s cancer treatment journey [19]. As students passed through various spaces, they were asked questions on general oncology or specific to that space’s specialty. Two studies described contouring modules involving medical students. One study compared a traditional didactic lecture with one that allowed an opportunity for students to use contouring software and found increased engagement and interest in RO with the inclusion of the interactive component [23]. The other described a live instructional session given by a resident or faculty member and compared students’ contours before and after the session with contours done by faculty [31].

Several studies described initiatives that aimed to teach trainees subjects outside of basic sciences. We identified 6 initiatives that taught or evaluated students about the multidisciplinary nature of oncology, including 2 that focused on teaching psychosocial topics in oncology [12,19,20,26,27,33]. Five of the 6 initiatives included some form of interactive learning, including role-playing or shadowing. It was felt that these paradigms were better-suited to help students appreciate the holistic approach to oncologic care.

We identified a single mentorship program described in 2 publications [21,22]. Established at a single institution, this mentorship program offered participants a clinical track, research track, or both. There were only 3 studies in our review that described interventions pertaining to research. Two described a single mentorship program with an option for a research track [21,22], while the other was a structured didactic and clinical shadowing experience for students in a pre-existing summer research program [27].

### 3.3. Study Characteristics

The majority of studies collected outcomes using surveys alone (n = 14). The most common comparison was pre- and post-intervention (n = 10), followed by no comparison (n = 6) and a comparison among participant groups (n = 4). Most studies employed a rating scale (n = 18), and the most common collected outcomes were related to program/intervention satisfaction (n = 17).

Two programs were the subject of multiple publications. Six studies reported on the Oncology Education Initiative, a vertically-integrated pre-clerkship and clerkship program incorporating didactic and interactive learning at Boston University [26]. Four studies reported findings from the Radiation Oncology Education Collaborative Study Group, which developed a standardized curriculum for clerkship students incorporating a targeted needs assessment and feedback and initially piloted the curriculum in 2012 [16].

### 3.4. Study Outcomes

#### 3.4.1. Educational Impact

There were 6 studies that examined participants’ knowledge before and after an intervention. The median increase in test grade was 9.5% (IQR: 7.7–15%). All studies reported statistical significance. Of these studies, 2 also examined participants’ knowledge between 2 intervention arms. One study compared knowledge after exposure to either an interactive contouring module or a standard didactic lecture, and found no statistical difference between the 2 groups. The other found that those who completed a structured RO clerkship program had improved scores compared to those that completed RO clerkships elsewhere (77.3% vs. 68.8%, *p* = 0.01).

#### 3.4.2. Impact on Career Decisions

Three studies examined the rates of participants who entered into a career in RO after being exposed to an intervention. The median rate was 29.3% (range: 4.12–30.5%). Two other studies measured the proportion of students interested in pursuing RO as a career, reporting rates of 25% and 89.1%. These data are strikingly higher compared to the national average of graduating medical students pursuing RO in Canada and the US, which is less than 1% [18].

## 4. Discussion

In this scoping review, we identified 25 primary articles pertaining to RO educational and career-planning initiatives in UME. Most interventions were a single session, involved clinical medical students, and based in North America. Didactic teaching was most commonly used, though a majority included interactive learning in addition to or in place of didactic teaching. As expected, there was heterogeneity across the outcomes reported, and most studies collected data using surveys alone. Six studies found an increase in test scores following an intervention, while 5 studies reported high rates of interest in pursuing RO compared to national averages. There was a paucity of studies reporting on formal mentorship programs and research programs.

Didactic lectures continue to be a popular method of instruction of RO topics in UME. However, data suggest that a typical adult learner’s attention span wanes after 15 to 20 min, and the information retained from passive lectures range from 5 to 30% [35]. There have been calls for more active learning strategies in order to improve learner engagement and retention [36]. Novel teaching methods may be more suited for certain aspects of RO teaching, such as role-playing to achieve learning objectives in the application and affective domains, or simulation for developing contouring skills. We identified 1 randomized trial that compared a traditional lecture to an interactive contouring module for pre-clinical medical students [23]. Although overall retention was not statistically different between the groups at 3 months, the interactive module participants had better knowledge of the radiation process and side effects, which are known knowledge gaps among medical students and primary care physicians [6]. We found that most interventions in our review used didactic lectures to introduce basic or foundational knowledge, whereas interactive components were used to improve learner engagement or knowledge retention.

Although RO teaching is known to be lacking throughout the world [5,6], 84% of studies that were included in our analysis were exclusively based in North America. There were only 2 studies from outside North America that described an educational or career-planning intervention specific to RO, 1 from Asia, and 1 from Europe [11,15]. Future studies describing interventions in other regions can help bring a global perspective to this issue.

Academic mentorship is an important aspect of a medical trainee’s personal and professional development. Potential benefits include improved research productivity, career satisfaction, and stress management. Much has been written on the topic, including commentary on how to structure and deliver mentoring in general and in RO [37,38]. However, we only identified 1 formal mentorship program described across two studies in our review. A recent scoping review regarding mentorship programs spanning all training levels in RO (medical students and residents) also demonstrated few published initiatives [39]. Most were dyad relationships and focused on resident mentorship. These gaps highlight the potential for future scholarship in both formal and informal mentorship initiatives in RO.

RO is a research-oriented specialty, attracting the highest percentage of medical student applicants with PhDs at 23% in 2014 in the US [40]. In addition, the mean number of research experiences among applicants increased from 3.7 to 6.1 from 2007 to 2018 [41]. Given the focus on research in RO, it was surprising that there were only 3 studies that pertained to medical student research. These studies reported outcomes related to program satisfaction and trainee productivity, but none described the structure of the research programs themselves. More literature describing effective program components can help inform the design or implementation of future research studentships.

Initiatives that reported on residency match rates had different aims depending on the status of RO residency in their respective locations. In Canada, it is uncommon for the number of medical student residency applicants to greatly exceed the number of RO residency positions. We identified 3 clinical elective programs in Canada whose primary aim was to increase medical student interest in RO residency [10,18,32]. All were geared towards pre-clinical students in an effort to introduce RO early in their medical training when most students decide on pursuing RO [22]. In contrast, RO is highly competitive in the US. One study reported on a mentorship program with a primary aim to give self-motivated applicants opportunities to network and improve their application to RO residency programs [22]. There is potential for expanding published experiences in interventions around the residency match process, especially those that target underrepresented groups in order to improve diversity in the field.

Strengths of this study include its systematic nature following published guidelines, its focus on studies reporting outcomes, its examination of the impact of interventions on both education and career interest, and its comprehensive overview of published interventions to inform new initiatives. However, this study is limited to articles published in English, and may not capture the entirety of the literature, especially outside of North America. We also recognize that this review may not represent all RO interventions for medical students that exist, as there may be successful or unsuccessful programs that have not been published.

## 5. Conclusions

There has been substantial growth in the number of RO initiatives for undergraduate medical students in recent years. The majority of interventions had a mix of traditional didactic teaching and interactive learning. Overall, the studies identified in this review demonstrate that RO initiatives directed towards medical students are effective at increasing knowledge of RO and generating career interest. Areas that may benefit from future studies include formal mentorship programs, interventions that aim to involve medical students in research, and interventions from institutions outside of North America.

## Figures and Tables

**Table 1:** Summary of review findings by data type.

Characteristic	N ^†^	%	Median	Range
Number of participants	24		96.5	16–682
Survey response rate	21		93.4%	53.8–100%
Target populations	25			
Clinical students ^‡^	12	48		
Pre-clinical students ^¶^	8	32		
All medical students	3	12		
Medical students and residents	1	4		
Trainees and staff	1	4		
Location	25			
North America	21	84		
Europe	2	8		
Multiple continents	1	4		
Asia	1	4		
Intervention objective	25			
Radiation oncology teaching	12	48		
General oncology teaching	5	20		
Career planning	4	16		
Basic science teaching	2	8		
Mentorship	2	8		
Intervention type	25			
Half-day course	13	52		
Clinical elective	3	12		
Multi-day course	3	12		
Mentorship program	2	8		
Summer program ^§^	2	8		
Game	1	4		
Shadowing	1	4		
Intervention duration	25			
Single session	15	60		
Less than 1 year	6	24		
Less than 1 month	2	8		
Less than 1 week	1	4		
Varied	1	4		
Outcome collection type	25			
Survey only	14	56		
Examination only	4	16		
Survey and examination	4	16		
Retrospective data collection	3	12		
Outcome comparison	25			
Pre- and post-intervention	10	40		
Statistically significant	9	90		
No comparison	6	24		
Among participant groups	4	16		
*Statistically significant*	*2*	*50*		
Between participants and non-participants	3	12		
*Statistically significant*	*2*	*67*		
Pre- and post-intervention and between groups	2	8		
*Statistically significant*	*2*	*100*		
Outcome methodology				
Rating scale	18			
Examination	8			
Qualitative	5			
Outcome type				
Program satisfaction	17			
Interest in radiation oncology	12			
Knowledge	9			
Teaching strategies				
Didactic lecture	17			
Interactive learning	13			
Clinical elective	6			
Shadowing	2			
Special topics				
Multidisciplinary nature of oncology	6			
Residency matching	4			
Research	3			
Psychosocial issues in oncology	2			
Contouring	2			

^†^ N = number of reporting studies. ^‡^ Clinical students are medical students in their third and fourth years. ^¶^ Pre-clinical students are medical students in their first and second years. ^§^ Summer programs can include a mix of didactic teaching, clinical work, mentorship, and/or research.

## Supplementary Materials

The following is available online at https://www.mdpi.com/1718-7729/28/1/72/s1, Table S1: Summary of study outcomes grouped by intervention objective.
