# Supplementary material for: A Scoping Review of Radiation Oncology Educational and Career-Planning Interventions in Undergraduate Medical Education
Source: Curr Oncol. 2021 Jan 31;28(1):740–9. doi: 10.3390/curroncol28010072 (PMC7985784; doi:10.3390/curroncol28010072)
Supplement: Supplementary file 1 [file curroncol-28-00072-s001.pdf]

Table S1. Summary of study outcomes grouped by intervention objective.

Intervention objective	Study	Target populations and number of participants	Intervention type	Outcome collection and comparison	Outcome summary
Radiation oncology (RO) teaching	Hirsch et al., 2007 (Oncology Education Initiative)	Clinical students‡, 149	Half-day course	Survey, no comparison	Participants were surveyed about program satisfaction and interest in RO. 56% of students felt there was not adequate exposure to oncology in clinical clerkship. 84% said they had no exposure or knowledge of radiation therapy prior to the program, while 88% reported they were motivated to learn after participating. 90% were satisfied or very satisfied with the session. 32% subsequently elected to participate in further RO teaching.
	Hirsch et al., 2008 (Oncology Education Initiative)	Clinical students, 153	Half-day course	Examination, pre- and post-intervention	Average test grade improved from 59% to 70% ($p = 0.011$).
	Hirsch et al., 2012 (Oncology Education Initiative)	Clinical students, 155	Half-day course	Examination, pre- and post-intervention	Average test grade improved from 62% to 68.9% ($p = 0.0001$).
	Zaorsky et al., 2012	Clinical students, 52	Clinical elective	Examination and survey, pre- and post-intervention	Participants were surveyed about program satisfaction. Average test grade improved from 64% to 82% ($p < 0.001$). Rating 1-9. Usefulness: 8.1, understanding of RO 8.8, recommendation of the rotation to their peers: 8.2.

Intervention objective	Study	Target populations and number of participants	Intervention type	Outcome collection and comparison	Outcome summary
	Golden et al., 2014 (ROECSG)	Clinical students, 18	Half-day course	Survey, no comparison	Participants were surveyed about program satisfaction. Scale 1-5. Rated 5 in usefulness to understand RO as a specialty, to increase student comfort in specialty decision and to help students with their transition to a RO residency.
	Agarwal et al., 2015 (Oncology Education Initiative)	Medical students, 169	Multi-day course	Survey, comparison among participant groups	Participants were surveyed about program satisfaction. Over 73% of students agreed or strongly agreed that the course met its learning objectives. Proportion of students who felt that the block was effective in preparing them to treat cancer patients in clerkship increased from 15.0% in 2010 to 45.8% in 2012. Women were more likely to view the course more positively ($p = 0.0234$) and consider it more effective ($p = 0.0303$).
	Golden et al., 2016 (ROECSG)	Clinical students, 88	Half-day course	Survey, pre- and post-intervention (in a single survey)	Participants were surveyed about program satisfaction and interest in RO. 89.1% of students reported intent to pursue RO as a specialty. Scale 1-5. Understanding RO: 5, specialty decision comfort: 4, help transition to RO residency: 4. Comfort with specialty decision was significantly higher after completing program (4 vs 5, $p < 0.001$).
	Gunther et al., 2016	Clinical students, 24	Half-day course (contouring)	Examination and survey, pre- and post-intervention	Participants were surveyed about program satisfaction. Scale 1-5. Importance: 4, improved comfort with contouring: 1 (pre) vs 4 (post). Mean Dice similarity coefficient improved from 0.29 to 0.68 ($p < 0.01$).

Intervention objective	Study	Target populations and number of participants	Intervention type	Outcome collection and comparison	Outcome summary
	Oskvarek et al., 2017 (ROECSG)	Clinical students, 109	Half-day course	Survey, comparison between participants and non-participants	Participants were surveyed about program satisfaction and interest in RO. Scale 1-5. Compared to radiation oncology applicants in non-curriculum sites, students in curriculum sites had greater confidence in radiation treatment decisions (4 vs 3, $p < 0.05$), knowledge of radiation biology (3 vs 2, $p < 0.01$), knowledge of radiation set-up (3 vs 2, $p < 0.05$) and knowledge of radiation treatment planning (3 vs 2, $p < 0.01$).
	Agarwal et al., 2018 (Oncology Education Initiative)	Clinical students, 495	Half-day course	Examination, pre- and post-intervention	Average test grade improved from 62% to 69.6% ($p < 0.001$).
	Neppala et al., 2018	Pre-clinical students¶, 43	Half-day course (contouring)	Examination and survey, pre- and post-intervention and between groups	Participants were surveyed about program satisfaction and interest in RO. Overall knowledge improved on the immediate post-test (87% vs 79%, $p = 0.007$). There were no significant differences between the didactic lecture and the contouring group. Scale 1-5. The contouring group had greater engagement (3.76 vs 3.10, $p = 0.02$), interest in completing a future clinical RO rotation (3.27 vs 2.2, $p = 0.01$).

Intervention objective	Study	Target populations and number of participants	Intervention type	Outcome collection and comparison	Outcome summary
	Golden et al., 2018 (ROECSG)	Clinical students, 146	Half-day course	Examination, pre- and post-intervention and between groups	Average test grade improved from 63.9% to 80.2% ($p < 0.01$). Compared to those who completed a non-ROECSG clerkship, those who completed ROECSG clerkship had higher scores (77.3% vs 68.8%, $p = 0.01$).
General oncology teaching	Finlay, 2001	Pre-clinical students, 45	Half-day course	Survey, no comparison	Participants were surveyed about knowledge and program satisfaction. Most (88%) returned positive comments on didactive lectures. The majority indicated a change in attitude after attending the session. Narrative comments were mostly related to increased awareness (65%) and empathy (30%).
	Mann et al., 2009	Clinical students, 186	Half-day course	Survey, no comparison	Participants were surveyed about program satisfaction. Scale 1-5. Average satisfaction: 3.85. Most valued session: role playing.
	Tsui et al., 2019	Pre-clinical students, 323	Shadowing	Survey, comparison among participant groups	Participants were surveyed about program satisfaction and interest in RO. 48% of students had greater appreciation for the multidisciplinary nature of oncology, 48% felt more competent interacting with oncologists, and 21% felt more competent with patients after attending session. Students were more likely to be interest in pursuing oncology with voluntary participation ($p = 0.02$). Involvement of faculty member was more favourably viewed ($p = 0.02$).

Intervention objective	Study	Target populations and number of participants	Intervention type	Outcome collection and comparison	Outcome summary
	Fukuchi et al., 2000	Clinical students, 16	Game	Examination and survey, pre- and post-intervention	Participants were examined before and after the intervention, and surveyed about program satisfaction. Significant linear relationship between number of questions answered correctly and number of games played ($R = 0.526$, $p < 0.001$). Post-game surveys were positive for appreciation of multidisciplinary nature of cancer management, knowledge of malignancies and understanding of oncologic principles (scale 1-5; 4.56, 4.50, 4.56).
	McKillip et al., 2017	Pre-clinical students, 33	Summer program[§]	Survey, pre- and post-intervention	Participants were surveyed about program satisfaction and interest in RO. Scale 1-5. Understanding of clinical oncology: 4 vs 2, $p < 0.01$. Understanding of research oncology: 4 vs 2, $p < 0.01$. Participants were more likely to talk about oncology as a continuum of care, includes survivorship, and screening/prevention in their post-program assessments.
Career planning	Barrett et al., 2008	Pre-clinical students, 105	Clinical elective	Retrospective data collection, comparison between participants and non-participants	Data were collected on students' interests in RO. 4.12% of program participants between 1971-1981 became radiation oncologists, compared to 0.7% of all post-MD trainees in 1989 ($p = 0.0091$).

Intervention objective	Study	Target populations and number of participants	Intervention type	Outcome collection and comparison	Outcome summary
	Tamaki et al., 2013	Medical students and residents, 531	Half-day course	Retrospective data collection, comparison among participant groups	An increasing proportion of program participants were members of the Japanese Society for Radiation Oncology (from 17.5% in 2004 to 26.4% in 2012).
	Haupt et al., 2020	Pre-clinical students, 37	Clinical elective	Survey, pre- and post-intervention	Participants were surveyed about interest in RO. Post-program, a greater proportion of participants listed RO as a top 3 career choice (25.0% vs 13.8%, p = 0.2396). The majority (64.8%) of participants stated their interest in RO increased after the program.
	Kang et al., 2020	Pre-clinical students, 31	Summer program	Survey, pre- and post-intervention (in a single survey)	Participants were surveyed about program satisfaction and interest in RO. Scale 1-5. Overall educational value: 4.8. Post-program, most students (76%) reported an increased interest in oncology. Average proportion of students who entered a RO career was 30.5%, compared to 0.7% in national residency matching statistics.
Basic science teaching	Oertel et al., 2019	Pre-clinical students, 682	Multi-day course	Survey, no comparison	Participants were surveyed about program satisfaction. Scale: 0-100 (0 being the best). Anatomy and Imaging: 9.3-15.4. Seminars, practical training, other faculty courses: 22.3-33.3.

Intervention objective	Study	Target populations and number of participants	Intervention type	Outcome collection and comparison	Outcome summary
	Zumwalt et al., 2007	Trainees and staff	Multi-day course	Survey, comparison among participant groups	Participants were surveyed about program satisfaction. Scale 1-10. Overall interest: 9.5-10. Quality: 9.5-10. Amount of new knowledge: 8.6-9.6. Faculty ranked relevance of the class to their work lower than medical students/residents (8.6 vs 9.8).
Mentorship	Boyd et al., 2019	Medical students, 76	Mentorship program	Survey, no comparison	Participants were surveyed about program satisfaction and interest in RO. 77.3% of participants reported the program strongly affected their career choice. 77.3% of participants reported their research experience strongly or moderately affected their career choice. 100% found that the program was effective or very effective. 81.8% reported that mentorship was extremely important to their career.
	Hirsch et al., 2014 (Oncology Education Initiative)	Medical students, 22	Mentorship program	Retrospective data collection, comparison between participants and non-participants	Data were collected about participants' interest in RO. Mentorship grew from 3 students in 2004 to 11 in 2013. Research productivity grew from 3 publications in 2007 to 14 in 2013. 29.3% of participants applied to RO residency and 100% matched (compared to a match rate of 85.1% nationally).

Abbreviations: RO = Radiation Oncology. ROECSG = Radiation Oncology Education Collaborative Study Group.

‡Clinical students are medical students in their third and fourth years.

¶Pre-clinical students are medical students in their first and second years.

[§]Summer programs can include a mix of didactic teaching, clinical work, mentorship, and/or research.
